# High sensitivity of asymmetric ^18^F-THK5351 PET abnormality in patients with corticobasal syndrome

**DOI:** 10.1038/s41598-023-39227-x

**Published:** 2023-07-27

**Authors:** Masanori Kurihara, Kenji Ishibashi, Tomoyasu Matsubara, Keiko Hatano, Ryoko Ihara, Mana Higashihara, Masashi Kameyama, Aya Midori Tokumaru, Katsuhiko Takeda, Yasushi Nishina, Kazutomi Kanemaru, Kenji Ishii, Atsushi Iwata

**Affiliations:** 1 Department of Neurology, Tokyo Metropolitan Institute for Geriatrics and Gerontology, 35-2, Sakaecho, Itabashi-ku, Tokyo, 173-0015 Japan; 2 Integrated Research Initiative for Living Well With Dementia, Tokyo Metropolitan Institute for Geriatrics and Gerontology, Tokyo, Japan; 3 Research Team for Neuroimaging, Tokyo Metropolitan Institute for Geriatrics and Gerontology, Tokyo, Japan; 4 Department of Diagnostic Radiology, Tokyo Metropolitan Institute for Geriatrics and Gerontology, Tokyo, Japan; 5 Bunkyo Cognitive Neuroscience Laboratory, Tokyo, Japan

**Keywords:** Neurology, Neurodegenerative diseases

## Abstract

Corticobasal syndrome (CBS) is characterized by symptoms related to the asymmetric involvement of the cerebral cortex and basal ganglia. However, early detection of asymmetric imaging abnormalities can be challenging. Previous studies reported asymmetric ^18^F-THK5351 PET abnormalities in CBS patients, but the sensitivity for detecting such abnormalities in larger patient samples, including early-stage cases, remains unclear. Patients clinically diagnosed with CBS were recruited. All patients displayed asymmetric symptoms in the cerebral cortex and basal ganglia. Asymmetric THK5351 PET abnormalities were determined through visual assessment. Brain MRI, perfusion SPECT, and dopamine transporter (DAT) SPECT results were retrospectively reviewed. The 15 patients had a median age of 72 years (59–86 years) and a disease duration of 2 years (0.5–7 years). Four patients met the probable and 11 met the possible CBS criteria according to Armstrong criteria at the time of PET examination. All patients, including early-stage cases, exhibited asymmetric tracer uptake contralateral to their symptom-dominant side in the cerebral cortex/subcortical white matter and striatum (100%). The sensitivity for detecting asymmetric imaging abnormalities contralateral to the symptom-dominant side was 86.7% for brain MRI, 81.8% for perfusion SPECT, and 90% for DAT SPECT. White matter volume reduction was observed in the subcortical region of the precentral gyrus with increased THK5351 uptake, occurring significantly more frequently than gray matter volume reduction. THK5351 PET may be a sensitive imaging technique for detecting asymmetric CBS pathologies, including those in early stages.

## Introduction

Corticobasal syndrome (CBS) manifests as asymmetric cortical and extrapyramidal symptoms^1^ and has an underlying pathology that includes corticobasal degeneration (CBD), progressive supranuclear palsy (PSP), Alzheimer's disease (AD), and frontotemporal lobar degeneration with TDP-43 immunoreactive inclusions (FTLD-TDP)^2,3^. CBD and PSP are responsible for most non-AD CBS cases^4^, which are neuropathologically distinguished by 4-repeat tau inclusions in neurons and glial cells, neuronal loss, and associated astrogliosis^5–7^.

Although there have been considerable recent advancements in fluid and imaging biomarkers for AD, there is a lack of diagnostic and disease-monitoring biomarkers for CBD and PSP^8^. Most PET tracers that identify tau inclusions in AD (3-repeat and 4-repeat tau) demonstrate weaker binding to 4-repeat tau inclusions^9^, and research on emerging tracers continues. While traditional imaging methods, such as brain structural MRI, can display brain atrophy related to the disease pathology in CBS patients^10^, some patients may not exhibit the typical asymmetric brain atrophy. To detect early disease pathologies for prompt diagnosis and disease monitoring, more sensitive imaging techniques are necessary.

^18^F-THK5351 was initially developed as a first-generation tau tracer^11^. However, researchers later found strong off-target binding to monoamine oxidase B (MAO-B)^12–14^, demonstrated by a 36.7–51.8% uptake reduction by MAO-B inhibitors on average (five mild cognitive impairment, two AD dementia, and one PSP), leading to the creation of second-generation tau tracers with reduced off-target binding^9^. In PSP, THK5351 uptake was reduced by MAO-B inhibitors completely in an autoradiography study^12^ and 69–89% in an in vivo human study^14^, suggesting that most tracer uptake was due to MAO-B. Physiologically, MAO-B is abundant in the striatum, thalamus, and brainstem^15,16^; pathologically, it is present in high concentrations in reactive astrocytes at astrogliosis lesion sites^17^. Although ^11^C-l-Deprenyl PET has been utilized to detect MAO-B/astrogliosis, ^18^F-THK5351 PET provides enhanced sensitivity^18^ and is now applied as a PET ligand for detecting astrogliosis in various neurological conditions^19–27^.

In previous research, Kikuchi et al. found asymmetric ^18^F-THK5351 PET tracer uptake in brain regions that corresponded to symptoms in five CBS patients who fulfilled modified Cambridge criteria^28^ and had a disease duration between 1.5 and 4.6 years^29^. This group later reported longitudinal data from five CBS patients and proposed that ^18^F-THK5351 PET could also be valuable for disease monitoring, predominantly reflecting astrogliosis^30^. Moreover, they observed a higher precentral gyrus tracer accumulation in seven CBS patients compared to PSP, AD, and healthy controls^31^. A single case study also revealed that ^18^F-THK5351 PET could detect astrogliosis in an affected brain region of a patient with CBD presenting with frontal behavioral-spatial syndrome (FBS)^32^. While these findings suggest that ^18^F-THK5351 PET has potential as a diagnostic and disease-monitoring biomarker, its application in CBS patients is limited, and its efficacy in detecting asymmetric abnormalities in a larger patient population, including those in earlier stages, is yet to be determined. Furthermore, no comparisons have been made between ^18^F-THK5351 PET results and those of other imaging tests.

In this study, we present the findings of ^18^F-THK5351 PET and additional imaging techniques in an expanded cohort of CBS patients, encompassing individuals in the early stages of the disease.

## Methods

### Subjects

Patients diagnosed with CBS were enrolled for the THK5351 PET study from January 2020 to March 2023. We conducted a retrospective review of the participants' medical records and included those with a clinical diagnosis of CBS as of February 2023. All patients exhibited progressively worsening asymmetric higher cortical and extrapyramidal symptoms, meeting either probable or possible CBS according to the Armstrong criteria^1^ at the time of PET examinations.

### PET image acquisition and analysis

^18^F-THK5351 was prepared at the Tokyo Metropolitan Institute for Geriatrics and Gerontology, and PET imaging followed a previously established protocol^16^. PET images were normalized using the cerebellar cortex as a reference region, with the uptake set to one (uptake ratio index [URI]). Images were visually assessed, and asymmetry was evaluated by two specialists who were blinded to the clinical information and interpreted the brain PET images (K. Ishibashi and K. Ishii). The tracer uptake in the striatum was quantified by Dr. View/LINUX version R2.0 (AJS, Tokyo, Japan) by setting symmetric region of interest (ROI) (M. Kurihara).

### Image acquisition and analysis of other images

The results of brain structural MRI, perfusion single photon emission tomography (SPECT) using ^123^I-IMP or ^99m^Tc-ECD, and dopamine transporter (DAT) SPECT using ^123^I-FP-CIT were retrospectively reviewed. The results of statistical analyses were available for all SPECT tests (eZIS or 3D-SSP for perfusion SPECT and DAT View for DAT SPECT). Gray and white matter volume reduction analyses (voxel-based morphometry) based on 3D T1-weighted images using SPM8 plus DARTEL using Voxel-based Specific Regional Analysis system for Alzheimer’s Disease (VSRAD) advance 2 software^33^ were unavailable in five patients with CBS. The asymmetry of the MRI and SPECT findings was evaluated visually with reference to these statistical analyses, when available. These evaluations were conducted by trained radiologists (A.M. Tokumaru and M. Kameyama) who were blinded to the PET results.

### Determination of amyloid positivity

For those who consented, amyloid PET imaging using [^11^C] Pittsburgh compound B (PiB) PET^34^ or cerebrospinal fluid (CSF) biomarker testing using ELISA^35^ was employed to estimate amyloid positivity, as reported previously. In cases with discordant results, amyloid PET emission tomography results were used.

### Statistical methods

Statistical analyses were conducted using the software program R version 4.0.3 (R Foundation for Statistical Computing, Vienna, Austria) and graphical interface EZR (Saitama Medical Center, Jichi Medical University, Saitama, Japan)^36^, or GraphPad Prism version 9 (GraphPad Software, San Diego, CA, USA). Missing data were addressed using a pairwise deletion approach. Categorical variables were expressed as percentages, and differences between groups were assessed using Fisher's exact test. Continuous variables were displayed as medians (full range). Statistical significance was set at p < 0.05.

### Ethics approval

This study received approval from the Ethics Committee of Tokyo Metropolitan Institute for Geriatrics and Gerontology and have been performed in accordance with the ethical standards laid down in the 1964 Declaration of Helsinki and its later amendments.

### Consent

Written informed consent for PET studies was obtained from each patient or their guardian.

## Results

### Baseline characteristics

Table 1 presents a summary of the patients' baseline characteristics. The 15 patients had a median age of 72 years (range: 59–86 years), with 7 (46.7%) being female and a disease duration (time interval from symptom onset) of 2 years (range: 0.5–7 years). The prevalence of documented signs included: limb-kinetic apraxia (86.7%), ideomotor apraxia (66.7%), alien limb (6.7%), cortical sensory deficits (46.7%), dyscalculia (20%), speech or language impairment (66.7%), frontal executive dysfunction (46.7%), visuospatial deficits (26.7%), limb rigidity or akinesia (86.7%), limb dystonia (40%), and limb myoclonus (33.3%). In all, 4 patients met the probable CBS criteria, while 11 fulfilled possible CBS criteria according to the Armstrong Criteria during the PET examination. Nine patients also fulfilled the modified Cambridge criteria^28^; however, the remaining patients did not meet the criteria at the time of PET examination due to an insufficient number of criteria met (n = 5) or unavailability of trials to rule out L-dopa responsiveness (n = 1). Twelve patients were amyloid-negative, and three were amyloid-positive. All patients underwent THK5351 PET and brain structural MRI, while brain perfusion SPECT and DAT SPECT were performed in 11 and 10 patients, respectively. MRI volume reduction analysis results were available for 10 of the 15 patients, and was unavailable in 5 due to lack of 3D T1-weighted images.

**Table 1:** Baseline characteristics and dominant side of symptoms and imaging abnormalities.

Baseline characteristics																	Dominant side of abnormality					
Case no.	Age (years old)	Sex	Disease duration (years)	Limb kinetic apraxia	Ideo-motor apraxia	Cortical sensory deficit	Alien limb	Limb rigidity akinesia	Limb dystonia	Limb myoclonus	Speech and language impairment	Armstrong criteria for CBS	Modified Cambridge criteria	Amyloid PET (PiB)	CSF Aβ42 (pg/mL)	CSF p-tau181 (pg/mL)	Symptom	THK cortex	MRI	Perfusion SPECT	THK striatum	DAT SPECT
1	62	M	1	+	+	+	−	−	+	+	−	Probable	−	n/a	488	36.5	L	R	R	R	R	R
2	78	F	2	+	−	+	+	+	+	+	+	Probable	+	n/a	1093	70.2	L	R	R	R	R	R
3	70	M	4	+	n/a	+	−	−	+	+	+	Probable	+	n/a	n/a	n/a	R	L	L	L	L	L
4	71	M	1	+	−	−	−	+	−	−	+	Possible	−	n/a	86	30.8	L	R	−	L	R	n/a
5	78	F	1.5	−	+	−	−	+	+	−	+	Possible	+	n/a	869	27.6	R	L	L	L	L	L
6	69	M	1	+	+	−	−	+	+	+	−	Possible	+	n/a	735	25.0	R	L	L	L	L	L
7	72	M	0.5	+	+	+	−	+	−	+	−	Probable	−*	n/a	720	38.8	R	L	R	n/a	−	n/a
8	59	M	1	+	+	−	−	+	−	−	+	Possible	+	−	830	34.9	R	L	L	L	L	L
9	79	M	3	−	+	−	−	+	−	−	+	Possible	−	−	725	< 25.0	L	R	R	R	R	R
10	80	F	3	+	n/a	+	−	+	−	−	−	Possible	+	−	744	36.2	L	R	R	−	R	n/a
11	76	M	7	+	−	n/a	−	+	−	−	+	Possible	−	−	334	30.7	R	L	L	n/a	L	L
12	59	F	3	+	+	−	−	+	+	−	+	Possible	+	−	926	49.4	R	L	L	L	L	n/a
13	85	F	2.5	+	+	−	−	+	−	−	−	Possible	−	−	919	46.6	L	R	R	n/a	R	R
14	64	F	2	+	+	+	−	+	−	−	+	Possible	+	−	807	30.8	L	R	R	n/a	R	n/a
15	86	F	4	+	+	+	−	+	−	−	+	Possible	+	+	142	159.0	R	L	L	L	L	−

*THK* ^18^F-THK5351 PET, *MRI* brain structural MRI, *DAT* dopamine transporter, *M* male, *F* female, *n/a* not available, *R* right, *L* left, − no laterality, *PiB* [^11^C] Pittsburgh compound B, *CSF* cerebrospinal fluid, *Aβ42* amyloid-beta 1–42, *p-tau181* tau phosphorylated at threonine 181. Abnormal CSF Aβ42 or p-tau181 values using predetermined institutional cutoffs (Aβ42: 500 pg/mL, p-tau181 50.0 pg/ml) are underlined. *Unresponsiveness to levodopa treatment was not evaluated.

### Concordance of asymmetric imaging abnormalities with symptom dominant sides

**Figure 1:**
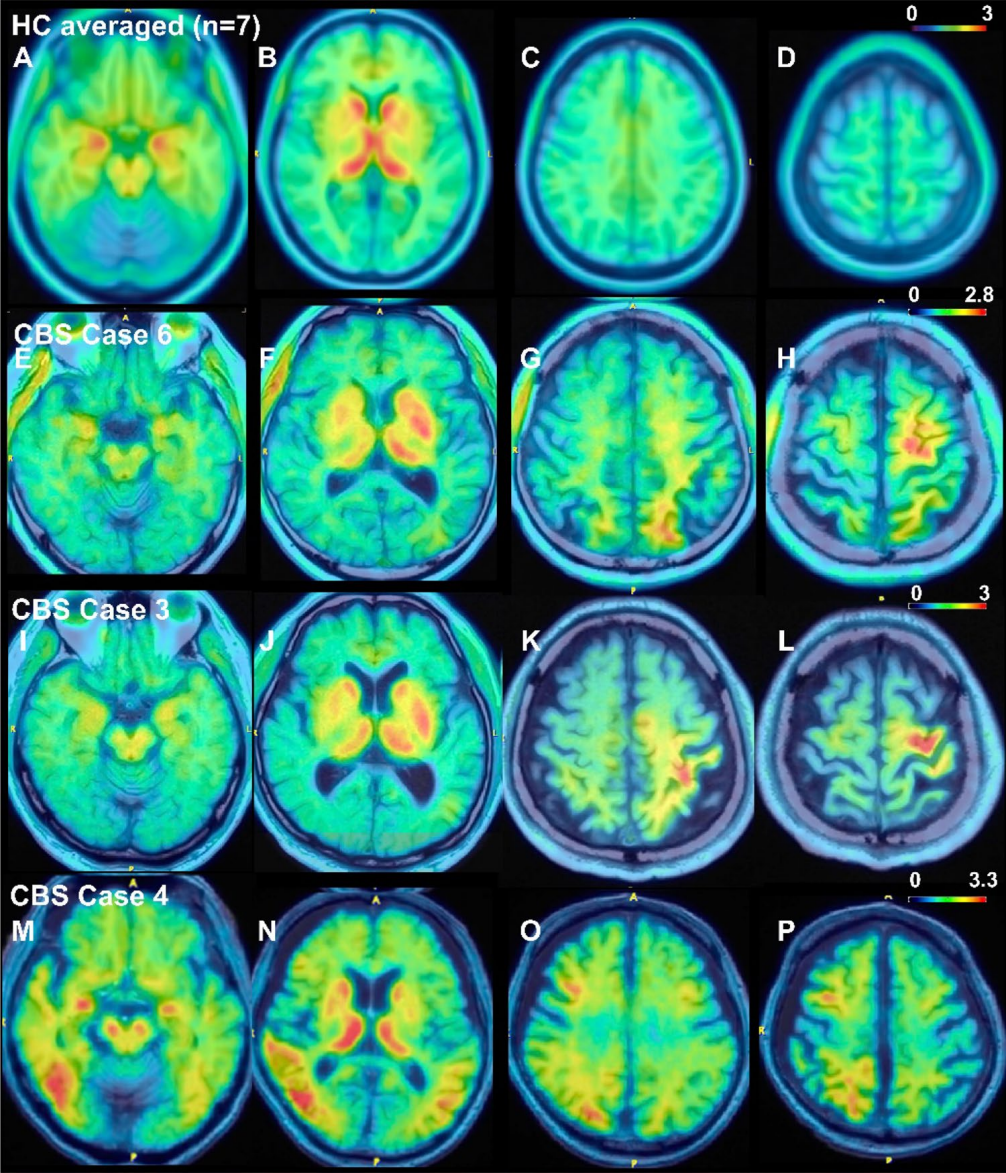
Representative ^18^F-THK5351 PET image results overlayed on structural brain MRI images. (**A**–**D**) Average results of seven healthy control volunteers. Physiological tracer uptake was primarily observed in the bilateral striatum, thalamus, and amygdala, where MAO-B is abundant. (**E**–**H**) Results for a 69-year-old man with possible CBS and a disease duration of one year (Case 6). The patient exhibited ideomotor apraxia, right dominant limb kinetic apraxia, rigidity, dystonia, and myoclonus. CSF biomarkers indicated non-AD. Left-dominated tracer uptake was observed in the frontal and parietal lobes, including the precentral gyrus. (**I**–**L**) Results for a 70-year-old man with probable CBS and a disease duration of 4 years (Case 3). The patient showed right-dominant limb kinetic apraxia, cortical sensory deficits, mild dysarthria, limb dystonia, and limb myoclonus. Left-dominated tracer uptake was observed in the frontal and parietal lobes, including the precentral and postcentral gyri. (**M**–**P**) Results of a 71-year-old man with possible CBS and a disease duration of a year (Case 4). The patient showed left-dominant limb kinetic apraxia and limb rigidity. CSF biomarkers suggestive of AD. Right-dominated tracer uptake was observed in the frontal, parietal, and temporal lobes. (**E**–**P**) Tracer uptake was observed in both the cerebral cortex and associated white matter. Striatal tracer uptake was higher contralateral to the symptom-dominant side in all patients. Color scales represent the uptake ratio index (URI), with the cerebellum as the reference region.

**Figure 2:**
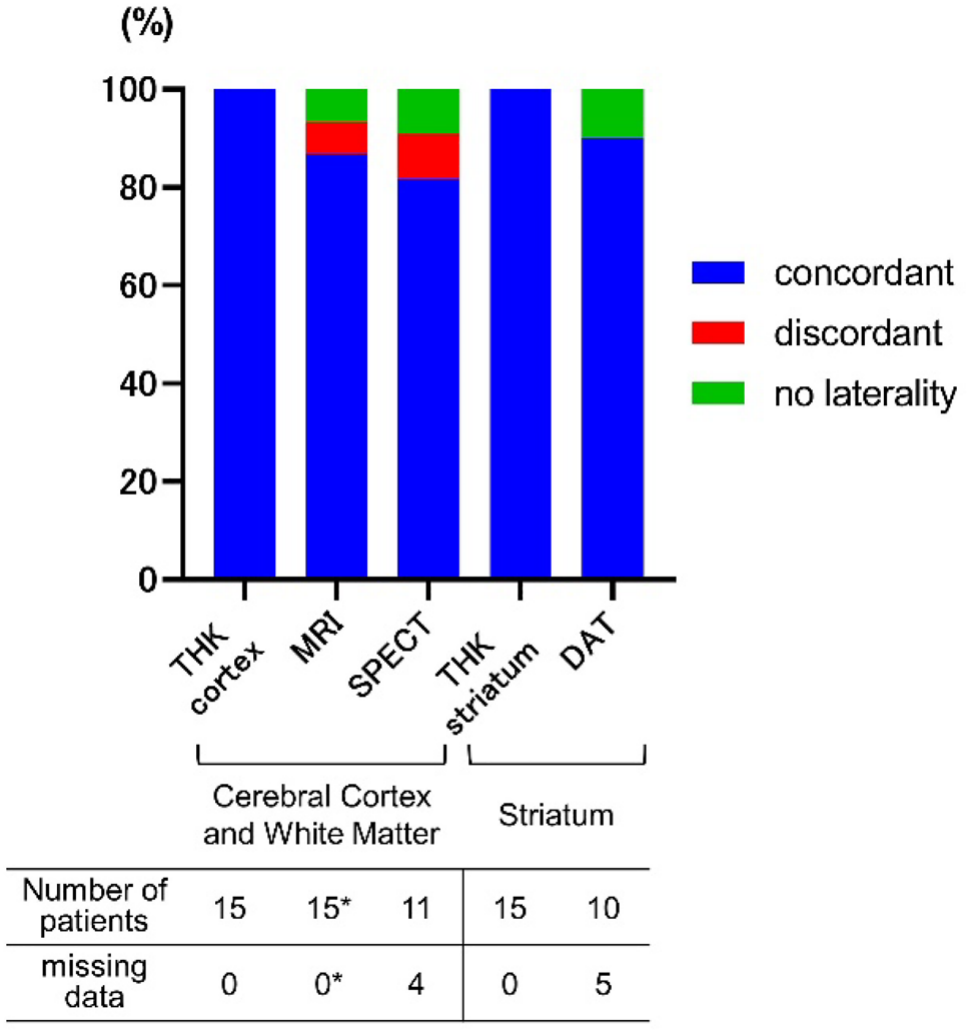
Concordance of imaging abnormality laterality against symptoms. The percentages of patients with asymmetric imaging abnormalities contralateral to the symptom-dominant side (concordant) are shown in blue. *Although brain MRI was available for all 15 patients, volumetric analyses based on 3D T1-weighted images were not available for 5 patients. Volumetric analyses were available for one patient with no laterality (CBS with CSF biomarker suggesting AD) and were unavailable for one patient with discordant results. *THK* ^18^F-THK5351 PET, *MRI* structural magnetic resonance imaging, *SPECT* brain perfusion single-photon emission tomography, *DAT* dopamine transporter SPECT.

Representative ^18^F-THK5351 PET images are provided in Fig. 1. All CBS patients exhibited asymmetric THK5351 uptake in the cerebral cortex and subcortical white matter, aligning with their symptoms. Asymmetric uptake was also observed in the striatum, corresponding to the laterality of symptoms in all patients (Supplementary Fig. 1). The concordance of the laterality of imaging abnormalities (contralateral to the symptom-dominant side) was 100% for THK5351 PET in both the cerebral cortex/subcortical white matter and striatum, 86.7% for brain structural MRI, 81.8% for brain perfusion SPECT, and 90% for DAT SPECT (Fig. 2).

Limb kinetic apraxia, ideomotor apraxia, cortical sensory deficits, and speech or language impairments were observed in 13, 10, 7, and 10 patients, respectively. THK5351 uptake was detected in the corresponding brain regions of most patients (Table 2).

**Table 2:** Cortical symptoms and correspondence with increased THK5351 uptake lesions.

	Number of patients	Corresponding brain lesions	Increased THK uptake	Brain atrophy by MRI	Decreased brain perfusion by SPECT
Limb-kinetic apraxia	13	Contralateral precentral gyrus	77% (10/13)	62% (8/13)	56% (5/9)
Ideomotor apraxia	10	Left fronto-parietal lobes	80% (8/10)	70% (7/10)	63% (5/8)
Cortical sensory deficits	7	Contralateral postcentral gyrus	71% (5/7)	43% (3/7)	60% (3/5)
Speech and language impairment	10	Left frontal lobe	90% (9/10)	70% (7/10)	63% (5/8)

*THK* ^18^F-THK5351 PET.

### Association between THK5351 uptake and volume reduction of gray and white matter at the precentral gyrus analyzed by MRI

**Figure 3:**
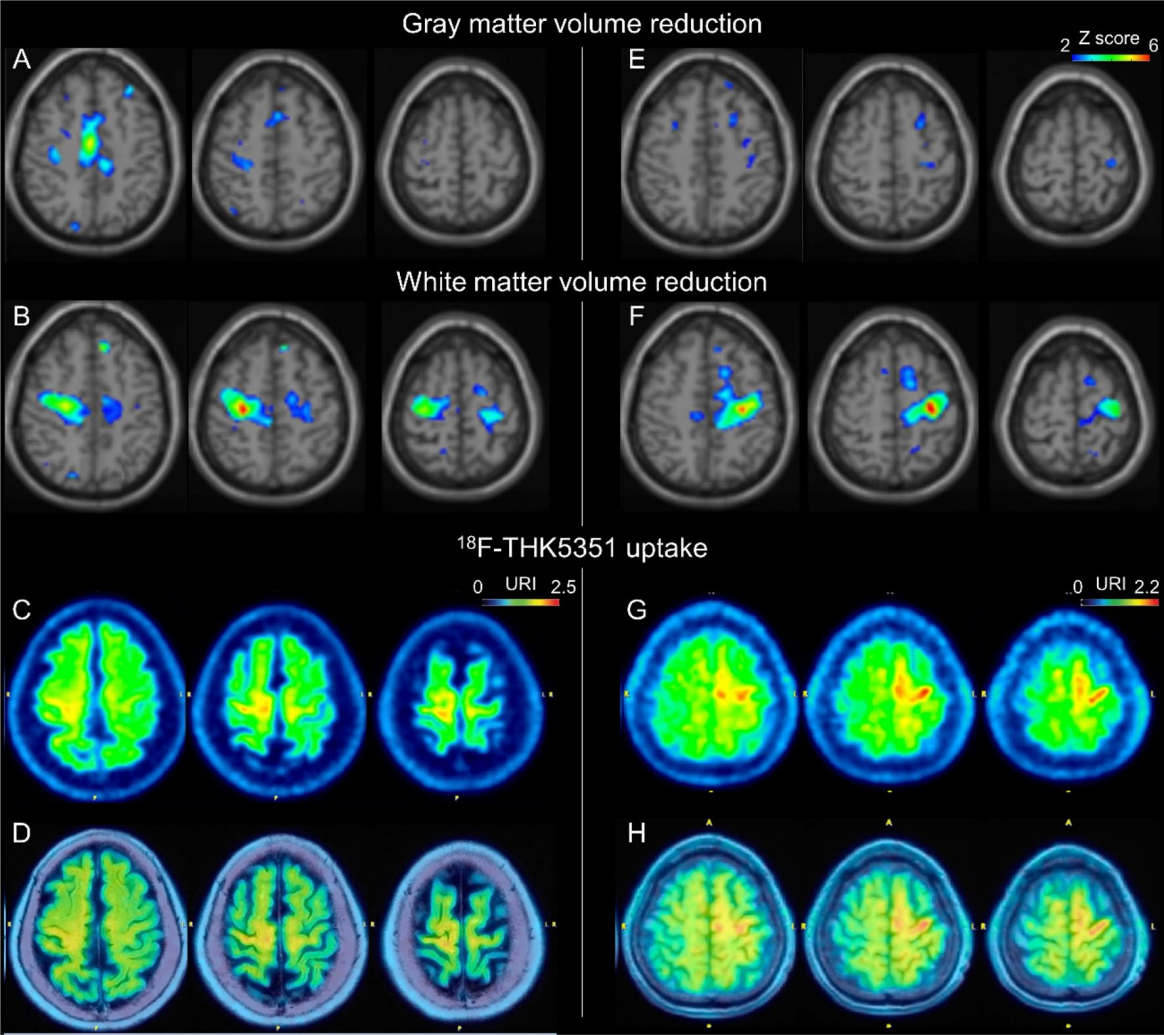
Representative relation between THK5351 uptake and gray or white matter volume reduction analyses. Imaging results for cases 10 (**A**–**D**) and 11 (**E**–**H**). The results of gray (**A**,**E**) and white matter (**B**,**F**) volume reduction analyses, ^18^F-THK5351 PET images (**C**,**G**), and ^18^F-THK5351 PET images overlaid on T1-weighted MRI (**D**,**H**) are shown. *URI* uptake ratio index with the cerebellum as the reference region.

THK5351 PET images overlaid on structural brain MRI images indicated that THK5351 uptake occurred in both the cerebral cortex and associated white matter (Fig. 1). Previous research has proposed that THK5351 uptake in the precentral gyrus is specific to CBS^31^, while we have previously reported that MRI white matter volume reduction analysis using SPM8 plus DARTEL is effective in detecting abnormalities in the frontal subcortical white matter around the precentral gyrus in CBS patients^37^. Thus, we assessed the relationship between THK5351 uptake and brain volume reduction around the precentral gyrus in patients with available 3D T1-weighted MRI images (n = 10). Images can be found in Fig. 3 and Supplementary Fig. 2.

Increased THK5351 uptake was observed in the right precentral gyrus in 6 patients (60%) and in the left precentral gyrus in 6 patients (60%). In these 12 precentral gyri, subcortical white matter volume reduction was suggested in 11 (91.7%) gyri, which was significantly more frequent than the gray matter volume reduction indicated in these gyri (3 [25%]; p = 0.00276).

## Discussion

We conducted THK5351 PET in 15 patients with CBS, including those in early stages. THK5351 PET identified asymmetric abnormalities contralateral to the symptom-dominant side with high sensitivity in both the cerebral cortex/subcortical white matter and the striatum.

The high sensitivity of asymmetric THK5351 PET abnormalities may contribute to early diagnosis of CBS. The diagnosis of CBS is based on clinical findings, and several diagnostic criteria have been proposed^1,28,38^. Although these diagnostic criteria are useful for diagnosis at advanced stages, their sensitivity may be insufficient in early stages^28^. The diagnostic criteria proposed in 2013 by Armstrong et al. included two sets of criteria for CBS with different diagnostic certainties: probable and possible^39^. Although using possible CBS criteria may aid in earlier diagnosis, its specificity is low, and incorporating imaging biomarkers may be necessary^39^.

Compared to other imaging techniques, THK5351 PET has several advantages. First, THK5351 PET exhibited a clear asymmetric abnormality recognizable through visual assessment, without the need for complex statistical image analysis. Second, THK5351 PET detected asymmetric abnormalities with higher concordance with (contralateral to) the laterality of the symptom-dominant sides compared to other imaging techniques. Third, THK 5351 PET identified asymmetric abnormalities in both the cerebral cortex/subcortical white matter and striatum, while other imaging techniques detected in either the cerebral cortex/subcortical white matter or striatum. Although still in research use and not specific for tau pathology, these characteristics may warrant future studies on THK5351 PET for the early diagnosis of CBS.

Another finding of this study was the correlation between THK5351 uptake in the precentral gyrus and white matter volume reduction in the subcortical region of the precentral gyrus, rather than gray matter volume reduction, as suggested by MRI voxel morphometry. CBD and atypical presentations of PSP (PSP-CBS) may display MRI signal abnormalities in the white matter corresponding to the disease pathology at autopsy^40–42^. White matter volume reduction analyses can reveal volume reductions in the frontal subcortical white matter around the precentral gyrus in patients with CBS^37^ and in other brain regions with other presentations of CBD^10^. A previous case report of a patient with CBD-FBS also suggested that the distribution of increased THK5351 uptake and white matter volume reduction were closely correlated in the frontal lobe, with reactive astrocytes observed along the corticomedullary junction in this brain region at autopsy^32^. Our findings indicate that astrogliosis and white matter volume reduction correlate in the subcortical region of the precentral gyrus in patients with CBS, although further studies with neuropathological assessments are needed to clarify the detailed distribution correlation between THK5351 and related pathologies (tau aggregates and astrogliosis) in both gray and white matter.

Astrogliosis is observed in affected brain regions across a wide range of neurodegenerative diseases. Recent evidence suggests that it may not be a simple downstream phenomenon of neurodegeneration but may partially contribute to disease progression in some diseases^43^. Although next-generation tau tracers specific for tau should have the advantage of visualizing the distribution of aggregated tau itself, combining the evaluation of the regional distribution and time course of both tau and astrogliosis in vivo may have the potential for further disease understanding^19,20^. Moreover, astrogliosis imaging may have the potential for disease monitoring, in combination with cerebrospinal and plasma GFAP concentrations, in future studies assessing disease-modifying therapies.

The neuropathological backgrounds of our patients with CBS are undetermined at this point. Previous autopsy studies have shown that patients with CBS have underlying pathology of CBD, PSP, AD, or rarely FTLD-TDP^2,3^. While twelve patients with CSF or amyloid PET results suggesting negative amyloid pathology likely have CBD or PSP, the underlying pathology of three with results suggesting positive amyloid pathology are difficult to determine. Case 1 showed slightly decreased CSF Aβ42 without memory impairment or increased uptake in medial temporal lobe. We assume that this patient has non-AD CBS with or without incidental amyloid pathology. Case 4 showed severely decreased CSF Aβ42 and the distribution of THK-5351 uptake was different from others (Fig. 1M–P) including medial and lateral temporal lobe. We assume that this patient has CBS-AD. Case 15 showed severely decreased CSF Aβ42 and increased CSF p-tau181. The patient showed memory impairment and increased uptake in medial temporal lobes, although lesions in the neocortex did not include lesions characteristic of CBS-AD such as lateral temporal lobes. We assume that this patient has non-AD CBS and mild cognitive impairment due to AD, although the possibility of CBS-AD remains.

This study has several limitations. First, neuropathological diagnosis was not available. Second, the sample size was small. Third, although previous studies have suggested that the majority of the increased THK5351 uptake represents MAO-B in non-AD patients^12–14^, and we excluded patients with AD by biomarkers in most patients, THK5351 uptake may represent both MAO-B and tau aggregates in some patients, especially in those with amyloid positivity. Future studies using a modified tracer ^18^F-SMBT-1 specific for MAO-B^44^ may resolve this issue. Fourth, while not only laterality but also the regions with increased THK5351 uptake corresponded to those expected from clinical symptoms, these concordances were not perfect. Follow-up studies are warranted to evaluate whether increased THK5351 uptake in these lesions will be observed later on.

In conclusion, THK5351 PET may be a sensitive imaging technique for detecting asymmetric CBS pathologies, including those in early stages.

## Supplementary Information

Supplementary Figures.

## Data Availability

The data sets and full protocol of the present study are available from the corresponding author on reasonable request.
