# Supplementary material for: High sensitivity of asymmetric 18F-THK5351 PET abnormality in patients with corticobasal syndrome
Source: Sci Rep. 2023 Jul 27;13:12147. doi: 10.1038/s41598-023-39227-x (PMC10374540; doi:10.1038/s41598-023-39227-x)
Supplement: Supplementary file 1 — Supplementary Figures. [file 41598_2023_39227_MOESM1_ESM.docx]

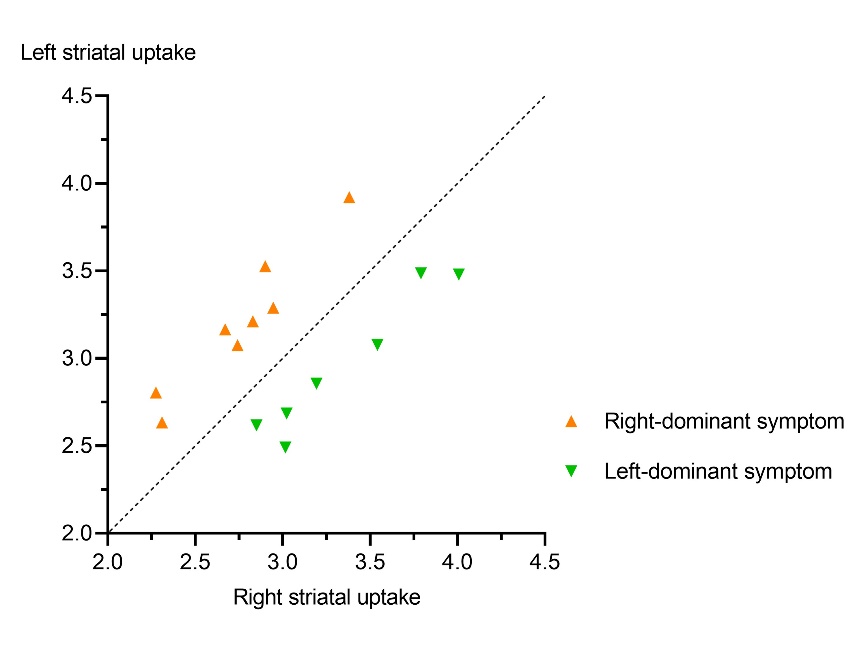


**Supplementary Figure 1. Quantification of THK5351 uptake in the striatum**

Mean uptake ratio index (URI) within symmetric region of interest (ROI) in right and left striatum are plotted for each patient. Patients with right-dominant symptom showed higher uptake in the left striatum and those with left-dominant symptom showed higher uptake in the right striatum. These asymmetries of quantification matched the result of visual assessment in all patients.


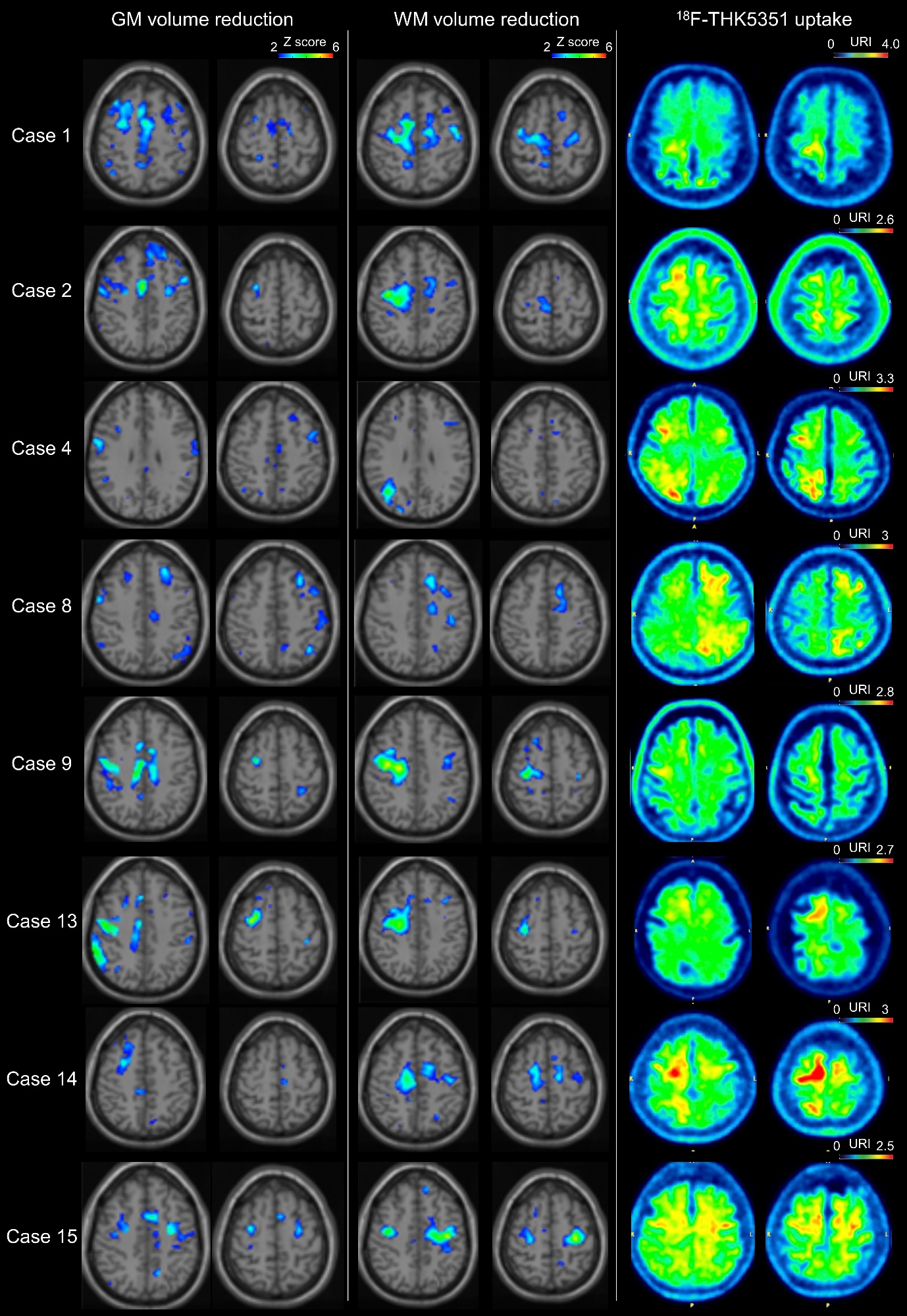


**Supplementary Figure 2. Relation between THK5351 uptake and gray or white matter volume reduction analyses**

The results of gray matter (GM) and white matter (WM) volume reduction analyses, and ^18^F-THK5351 PET images are shown for each patient. URI: uptake ratio index with the cerebellum as the reference region.
